# Exometabolom analysis of breast cancer cell lines: Metabolic signature

**DOI:** 10.1038/srep13374

**Published:** 2015-08-21

**Authors:** Lucas Willmann, Thalia Erbes, Sebastian Halbach, Tilman Brummer, Markus Jäger, Marc Hirschfeld, Tanja Fehm, Hans Neubauer, Elmar Stickeler, Bernd Kammerer

**Affiliations:** 1 Center for Biological Systems Analysis ZBSA, Albert-Ludwigs-University Freiburg, 79104 Freiburg, Germany; 2 University Medical Center Freiburg, 79106 Freiburg, Germany; 3 Institute of Molecular Medicine and Cell Research, Albert-Ludwigs-University Freiburg, 79104 Freiburg, Germany; 4 Institute of Biology II, Albert-Ludwigs-University Freiburg, 79104 Freiburg, Germany; 5 Universitätsfrauenklinik der Heinrich-Heine-Universität Düsseldorf, Moorenstr. 5, 40225 Düsseldorf, Germany; 6 Institute for Biology III, Faculty of Biology, Albert-Ludwigs-University Freiburg, Germany; 7 Spemann Graduate School of Biology and Medicine, Albert-Ludwigs-University Freiburg, Germany; 8 Department of Obstetrics and Gynecology, University Hospital Freiburg, Freiburg 79106, Germany; 9 German Cancer Consortium (DKTK), Heidelberg, Germany; 10 German Cancer Research Center (DKFZ), Heidelberg, Germany

## Abstract

Cancer cells show characteristic effects on cellular turnover and DNA/RNA modifications leading to elevated levels of excreted modified nucleosides. We investigated the molecular signature of different subtypes of breast cancer cell lines and the breast epithelial cell line MCF-10A. Prepurification of cell culture supernatants was performed by cis-diol specific affinity chromatography using boronate-derivatized polyacrylamide gel. Samples were analyzed by application of reversed phase chromatography coupled to a triple quadrupole mass spectrometer. Collectively, we determined 23 compounds from RNA metabolism, two from purine metabolism, five from polyamine/methionine cycle, one from histidine metabolism and two from nicotinate and nicotinamide metabolism. We observed major differences of metabolite excretion pattern between the breast cancer cell lines and MCF-10A, just as well as between the different breast cancer cell lines themselves. Differences in metabolite excretion resulting from cancerous metabolism can be integrated into altered processes on the cellular level. Modified nucleosides have great potential as biomarkers in due consideration of the heterogeneity of breast cancer that is reflected by the different molecular subtypes of breast cancer. Our data suggests that the metabolic signature of breast cancer cell lines might be a more subtype-specific tool to predict breast cancer, rather than a universal approach.

Breast cancer is the most frequently diagnosed type of cancer and the leading cause of death by cancer among females. Twenty-three percent of all cancer cases are breast cancer cases and 14% of all deaths by cancer can be traced back to breast cancer^1^.

Besides the analysis of genomic and proteomic profiles, the understanding of biochemical processes based on metabolites is of particular importance in order to find characteristic biomarkers for breast cancer. Tumor markers can be produced by cancer cells or by healthy cells as a reaction to the disease. This markers can be single-protein-, RNA-, DNA-based markers as well as a molecular signature consisting of multiple compounds^2^.

The tumor-associated antigens CEA (Carcinoembryonic antigen) and CA (Carbohydrate antigen) 15-3 have been discussed as biomarkers for breast cancer progression, but are not recommended for the early diagnosis and therapy surveillance of cancer^3^.

The altered RNA metabolism of cancer cells results in elevated excretion levels of modified nucleosides in different biological fluids. It has been reported that the tRNA turnover rate in tumor tissue exceeds the tRNA turnover rate in normal tissue resulting in rapid degradation and excretion of modified nucleosides^4^. As an explanation for differences of base composition in tumor tRNA numerous reasons have been discussed, such as changes in tRNA concentration, presence of tRNA with altered sequences and aberrant modifications^5^. Regarding this phenomenon blood^6^, urine^7^,^8^,^9^ and supernatants of breast cancer cell lines^10^ have been analyzed in order to find preferably specific and sensitive biomarkers for the early diagnosis of breast cancer.

Nucleosides consist of a ribose moiety bound to a nucleobase via beta-glycosidic linkage. The common ribonucleosides adenosine, guanosine, uridine and cytidine as well as modified nucleosides are components of RNA. In the nucleolus, RNA can be modified post-transcriptionally by several enzymes resulting in modifications like methylation, hydroxylation, reduction, isomerization, sulfur/oxygen substitution or addition of sidechains^11^. Today over 100 modified nucleosides are known, present in different RNA types, such as tRNA, mRNA, rRNA and snRNA^12^. In general, all RNA types contain modifications, but tRNA is by far the most-modified RNA type regarding to extent and diversity of modifications. Modified RNA is degraded to modified nucleosides in the cytoplasm by nucleases, phosphodiesterases and phosphatases. Adenosine, guanosine, uridine and cytidine (Fig. 1) are phosphorylated, resulting in ribose-1-phosphate and the corresponding nucleobase. Afterwards the nucleobase is recycled to adenosine triphosphate (ATP), guanosine triphosphate (GTP), uridine triphosphate (UTP) or cytidine triphosphate (CTP) in the salvage pathway (Fig. 1) and returned into the nucleus. Alternatively, unmodified nucleosides can be excreted out of the cell and metabolized to uric acid, CO_2_, NH_3_, β-Aminoisobutyrate or β-Alanine. Due to the lack of specific kinases for synthesis of modified nucleoside triphosphates in mammalian cells, modified nucleosides do not enter the salvage pathway for RNA rebuilding and therefore they are excreted quantitatively as metabolic endproducts. Consequently, the insertion of modified nucleoside triphosphates into inappropriate positions in tRNA or rRNA is avoided^13^. In Fig. 2, some modified nucleosides are depicted.

Though the exclusion of interferences during excretion, e.g. enzymatic modifications by blood components or liver secretions or contamination by bacterial metabolites of the intestinal flora, the analysis of supernatants of breast cancer cell lines provides an unaltered metabolic signature. The utilization of two dimensional monolayer cell lines delivers an experimental *in vitro* tumor model with standardized format and an excellent possibility of modelling proliferation and signalling pathways. But the heterogeneity of cancer cannot be reflected by a single cell line because a single cell line does not consider complex interactions between cell types and tissue microenvironment^14^.

Breast cancer cells can be divided into five different molecular subgroups: Luminal A, Luminal B, HER2 (Human epidermal growth factor receptor 2) over-expression, Basal-like and Claudin-low. Originally, this sub classification has been based on gene expression analysis^15^, but has recently been reduced to the determination of ER (Estrogen receptor), PR (Progesterone receptor), expression of the proliferation marker Ki-67 and HER2 status by immunohistochemistry and FisH (Fluorescence in situ hybridization)^16^. Luminal A breast cancer is better differentiated, sensitive to chemotherapy and endocrine therapy, exhibits a low Ki-67 level and low proliferation gene cluster as well as good prognosis for a cancer patient. Luminal B breast cancer is mainly resistant to chemotherapy, less sensitive to endocrine therapy, shows a high expression of proliferation gene cluster and Ki-67 connected with poor prognosis for the cancer patient^17^,^18^,^19^. The proliferation gene cluster contains several genes linked to the cell cycle progression and known as proliferation markers, such as CCNB1 (Cyclin B1) and MKI67^20^. Claudin-low breast cancers are invasive ductal carcinomas that exhibit cancer stem cell like features, molecular markers for epithelial-to-mesenchymal transition and immune response genes. The expression of luminal differentiation markers is low to absent, whereas the frequency of metaplastic and medullary differentiation is elevated^21^. Breast cancer with HER2 over-expression results in a poor prognosis for a breast cancer patient and is responsive for trastuzumab and chemotherapy. Breast cancer is a very heterogeneous disease and different breast cancer cell lines should reflect this biological diversity.

Nucleosides have been analyzed by numerous methods before, e.g. high performance liquid chromatography (HPLC) coupled to time of flight (TOF) MS via electrospray ionization (ESI)^22^, liquid chromatography (LC) ion trap (IT) MS^23^, ESI Fourier transform ion cyclotron resonance (FTICR) MS^8^ or matrix assisted laser desorption/ionization (MALDI) TOF-MS^24^.

Despite numerous approaches there is no consistent metabolic signature consisting of compounds from RNA metabolism, which can clearly be interrelated with breast cancer. To the authors’ best knowledge a subtype specific approach has never been executed. The aim of this study was to investigate the excretion of compounds from RNA metabolism and cross-linked pathways in cancerously altered metabolism in comparison to the breast epithelial cell line MCF-10A and to compare the metabolic signature of different sub classifications of breast cancer cell lines.

## Methods

### Chemicals

We used methanol (LC/MS grade, Carl Roth GmbH & CO, Karlsruhe, Germany), ultrapure water prepared from Purelab Ultra laboratory water purification system (Elga LabWater, Celle, Germany) and analytical grade formic acid (Sigma-Aldrich, Munich, Germany) for HPLC analysis. Ammoniumacetat (VWR international, Darmstadt, Germany), sodium chloride (Carl Roth GmbH & CO, Karlsruhe, Germany) and boronate-derivatized polyacrilamide gel (Biorad, Richmond, USA) were used for affinity chromatography. S-Adenosylmethionine (SAM), S-Adenosylhomocysteine (SAH), Cytidine (C), Adenosine (A), N^4^-Acetylcytidine (ac^4^C), Uridine (U), Guanosine (G), 5,6-Dihydrouridine (D), 5-Hydroxyuridine (5-OHU), 5-Aminoimidazol-4-carboxamid 1-β-D-Ribofuranosid (AICAR), Inosine (I), 5-Methyluridine (m^5^U), 1-Methyladenosine (m^1^A), N^6^-Methyladenosine (m^6^A), 1-Methylguanosine (m^1^G), N^2^-Methylguanosine (m^2^G), 5-Desoxy-5-Methylthioadenosine (MTA), Xanthosine Dihydrate (X) and Nucleoside Test Mix were purchased from Sigma-Aldrich (Munich, Germany). Pseudouridine (Ψ) and Isoguanosine (Isog) were purchased from Carbosynth (Berkshire, UK). N^2^,N^2^,7-Trimethylguanosine (TMG) was purchased from Biolog (Bremen, Germany).

### Cell culture and culture conditions

The starting cultures of the breast cancer cell lines BT-474, MDA-MB-231 and MD-MBA-453 were kindly donated by Prof. Stickeler of University Hospital Freiburg, Germany. MDA-MB-453 was grown in RPMI 1640 (Life Technologies, Darmstadt, Germany)/10% Newborn Calf Serum (Life Technologies, Darmstadt, Germany)/1% Penicilline/Streptomycine (Life Technologies, Darmstadt, Germany)/1% HEPES (Life Technologies, Darmstadt, Germany). BT-474 and MDA-MB-231 were grown in DMEM F12 (Sigma-Aldrich, Munich, Germany)/10% Newborn Calf Serum (Life Technologies, Darmstadt, Germany)/1% Penicilline/Streptomycine (Life Technologies, Darmstadt, Germany)/1% HEPES (Life Technologies, Darmstadt, Germany). The starting culture of the reference cell line MCF-10A was provided by Gillian Lehrbach and Roger Daly, Garvan Institute of Medical Research, Sydney. MCF-10A was grown in DMEM F12 (PAN-Biotech GmbH, Aidenbach, Germany)/5% Horse Serum (PAA, Cölbe, Germany)/1% Glutamine (PAN-Biotech GmbH, Aidenbach, Germany)/1% HEPES (PAN-Biotech GmbH, Aidenbach, Germany)/1% Penicilline/Streptomycine (PAN-Biotech GmbH, Aidenbach, Germany)/250 μg Hydrocortisone (Sigma-Aldrich, Munich, Germany)/50 μg Choleratoxin (Sigma-Aldrich, Munich, Germany)/10 μg Epidermal Growth Factor (R&D Systems, Wiesbaden-Nordenstadt, Germany)/4.858 mg Insuline (Novo Nordisk Pharma GmbH, Mainz, Germany).

Cells were seeded in 75 cm^2^ plastic flasks (Greiner Bio One GmbH, Frickenhausen, Germany) and grown at 37 °C/5% CO_2_. After reaching confluence, cells were washed with 8 ml 1x PBS (Life Technologies, Darmstadt, Germany) twice and incubated with 5 ml 1x Trypsin/EDTA (Life Technologies, Darmstadt, Germany) at 37 °C/5% CO_2_. Trypsinization was stopped by adding medium with serum. To check the number of living cells 10 μl cell suspension was mixed with 40 μl Trypan Blue solution, consisting of 0.5% Trypan Blue (Sigma-Aldrich, Munich, Germany) and 0.9% NaCl in double-destilled water, and incubated for 5 min at 37 °C/5% CO_2_. The number of living cells was averaged using a Neubauer counting chamber under a light microscope. Subsequently cells were centrifugated (400 g, 5 min, 4 °C) in order to remove the serum-containing medium. Afterwards defined numbers of cells were seeded in three flasks for each cell line using the proper media containing additives without serum. After four days of incubation (37 °C/5% CO_2_) the supernatant was collected and centrifugated (1000 g, 10 min, 4 °C) to remove cell debris. The number of living cells was averaged again using a Neubauer counting chamber. The supernatant was stored at −20 °C.

### Characteristics of investigated cell lines

The characteristics of the investigated breast cancer cell lines are summarized in Table 1. The reference cell line MCF-10A is a spontaneously immortalized human breast epithelial cell line derived from mastectomy tissue from a 36 year old, premenopausal woman with fibrocystic disease. MCF-10A has characteristics of normal breast epithelium by numerous criteria^25^. It has been shown that MCF-10A cells are mostly ER negative, PR negative and belong to the Basal-like subtype^17^.

### Extraction of cis-Diols

Before the HPLC separation the cell culture supernatants were purified by cis-diol specific affinity chromatography using boronate-derivatized polyacrylamide gel. The method was developed by Liebich *et al.*^26^. 50 ml of cell culture supernatant were spiked with an internal standard solution (0.25 mM isoguanosine), alkanalized to pH 8.8 with ammonia solution and put on a column (column volume: 200 ml; column diameter 15 mm) with 500 mg boronate-derivatized polyacrylamide gel. Washing was achieved with 25 ml ammonium acetate solution (0.25 M, pH 8.8) and 6 ml methanol/water (1:1, v/v). The cis-diols were eluted with 50 ml 0.2 M formic acid in methanol/water (1:1, v/v). The solvent was removed by evaporation and the pellet was dissolved in water. Thereby the eluted fraction was concentrated 50-fold. 10 μl of the residues were injected for HPLC analysis.

### Instrumentation

The HPLC analysis was performed on an Agilent 1200 system (Agilent, Waldbronn, Germany) consisting of a degaser (G1379 B), a binary pump (G1329 A), an autosampler (G1329 A), a column oven (G1316 A) and a VWD (G1314 B). Reversed-phase chromatography was carried out on an Eclipse XDB-C18 (5 μm, 150 mm × 4,6 mm) column (Agilent, Waldbronn, Germany) with a solvent gradient of water + 0.5% formic acid (A) and methanol + 0,5% formic acid (B) at a flow rate of 0.5 ml/min. The column was operated at 20 °C. The following gradient was used: 0–15 min, 2 to 30% B; 15.1–25 min, 50% B; 26–29 min, 90% B; 30–35 min: 2% B.

Mass spectra were acquired on an Agilent 6460 triple quadrupol mass spectrometer (Agilent, Waldbronn, Germany) equipped with an electrospray ionization source (ESI Jet Stream). The following MS settings were applied: capillary voltage: 3500 V, nozzle voltage: 500 V, gas temperature: 300 °C (flow rate: 5 l/min), sheath gas: 250 °C (flow rate: 11 l/min), nebulizer pressure: 45 psi. All scan modi were carried out in the positive ionization mode with a step size of 0.1 amu, a threshold of 0 and a cell accelerator voltage of 7 V. The data storage was centroid. Nitrogen was used as collision gas.

For purchased standard substances the fragmentor voltage and collision potential for the multiple reaction monitoring (MRM) was determined using the Mass Hunter Optimizer Software (Agilent, Waldbronn, Germany) (see Electronic Supplementary Material). Compounds, that were not available as standard substance, were detected in fullscan, neutral loss scan and product ion scan (see Electronic Supplementary Material). The fragmentation pattern was compared to databases^12^,^27^,^28^ and literature^8^,^10^,^29^. Based on product ion scans with different fragmentor voltage and collision potential appropriate values were set for MRM operating mode. For the semi-quantitative analysis MRM was executed. The applied fragmentor voltages and collision potentials are displayed in Tab. 1. The dwell time was set to 5 ms and the cell accelerator voltage was set to 7 V.

For post processing Agilent MassHunter Qualitative Analysis B.04.00 and Agilent MassHunter Quantitative Analysis B.05.00 were used. Peaks with a minimal signal-to-noise ratio of 1:5 and a maximal retention time difference of 0.4 min were used for the semi-quantitative analysis. For the semi-quantitative analysis the peak area of each analyte was divided by the peak area of the internal standard isoguanosine and multiplied by factor 1000. Therefore the quantifier MRM transitions were utilized. The ascertained values for compounds resulting from culture media without cell treatment were subtracted. The resulting values were normalized using the counted number of cells at the moment of supernatant sampling. Statistical analysis was executed using Microsoft Excel 2007 and MetaboAnalyst 2.0^30^. Partial Least Squares Discriminant Analysis (PLSDA), leave one out cross-validation with selection based on Q^2^ and Variable Importance Projection (VIP) score were generated in MetaboAnalyst. Two-dimensional hierarchical cluster analysis was performed in MetaboAnalyst using the normalized peak areas. Peak areas were mean-centered and divided by the standard deviation of each variable. Pearson’s correlation coefficient was used for distance measurements and Ward’s minimum variance was used as a clustering method.

## Results and Discussion

In the present study we analyzed the differences of the metabolite excretion of the breast cancer cell lines MDA-MB-453, MDA-MB-231, BT-474 and the breast epithelial cell line MCF-10A using LC-MS.

The averaged cell numbers of the examined cell lines at seeding and post harvesting lead to average doubling times for MCF-10A, MDA-MB-231, MDA-MB-453 and BT-474, which were 27.0 +/−0.3, 23.7 +/− 2.5, 20.4 +/− 1.2 and 26.2 +/− 1.6 h (mean +/− standard deviation), respectively. Similar doubling rates have been determined before^31^,^32^,^33^,^34^.

**Figure 1:**
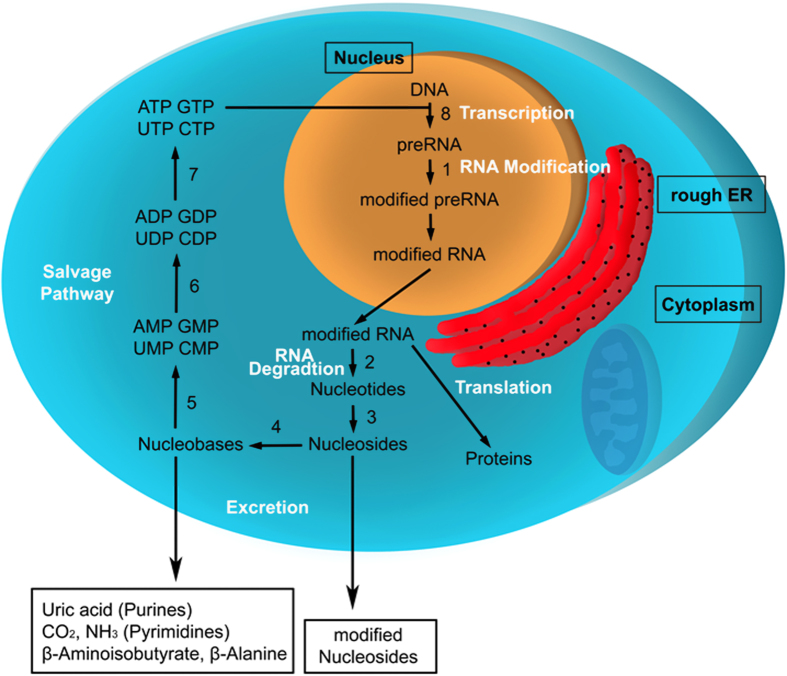
Cellular RNA-metabolism (Abbrevations: DNA = Deoxyribonucleic acid; RNA = Ribonucleic acid; ER = Endoplasmatic reticulum; A-, G-, C-, UMP = Adenosine-, Guanosine-, Cytidine-, Uridine-mononucleotide; Involved enzymes: 1) e.g. RNA-Methyltransferases, 2) Nucleases, Phosphodiesterases (EC: 3.1.4) 3) Phosphatases (EC: 3.1.3) 4) Phosphorylases (EC: 2.4.1.1) 5) Nucleoside-phosphoribosyltransferases (EC: 2.7.1.48; EC: 2.7.1.20) 6) Nucleosidephosphatekinases (EC: 2.7.4.3; EC: 2.7.4.14) 7) Nucleoside-diphosphatekinases (EC: 2.7.4.10; EC: 2.7.4.6) 8) Helicase (EC: 3.6.4.12), DNA-Polymerase (EC: 2.7.7.7), DNA-Ligase (EC: 6.5.1.1); this figure has been drawn by LW).

In order to identify the compounds of different cell culture supernatants, we performed neutral loss scans, product ion scans and fullscans. The decay into a neutral sugar moiety and the nucleic base fragment at the fragile N-glycosidic bond is characteristic for nucleosides. The CNL of 132 amu is a mark for occurrence of an unmodified ribose moiety, whereas the CNL of 162 amu (MTA), 178 amu (5-Deoxy-5-Methylthioadenosine-sulfoxide (MTA-SO)), 249 amu (SAH) and 263 amu (SAM) indicate the occurrence of a modified ribose moiety (Electronic Supplementary Material Fig. 1). Nucleosides without the labile C-N-glycosidic bond, e.g. the nucleoside Ψ with a stable C-glycosidic bond, do not provide characteristic CNL chromatograms.

**Figure 2:**
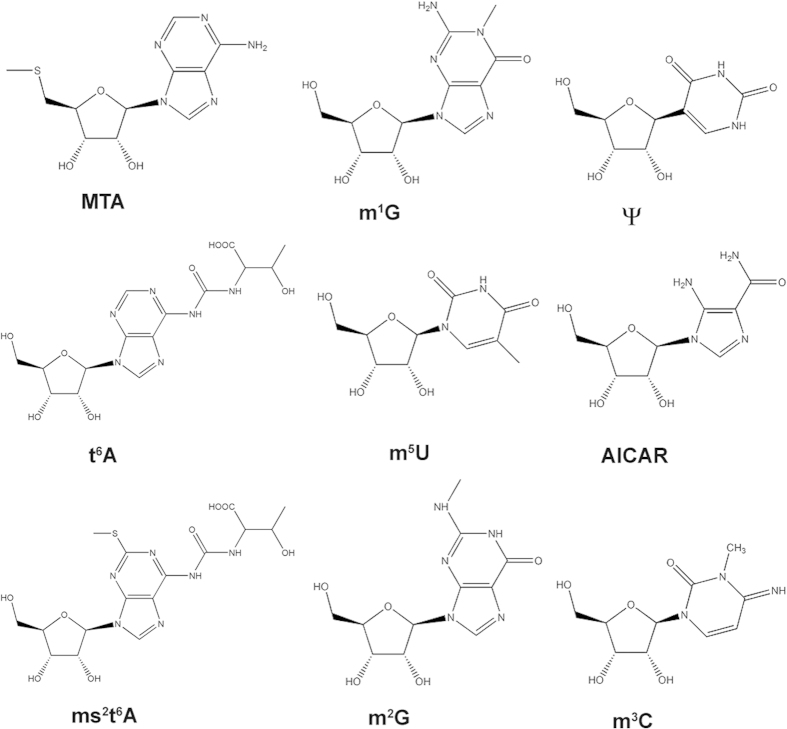
Structures of some exemplary target compounds (MTA = 5-Desoxy-5-methylthioadenosine, m^1^G = 1-Methylguanosine, Ψ = Pseudouridine, t^6^A = N^6^-Threonylcarbamoyladenosine, m^5^U = 5-Methyluridine, AICAR = 5-Aminoimidazol-4-carboxamid 1-β-D-Ribofuranosid, ms^2^t^6^a = 2-Methylthio-N^6^-threonylcarbamoyladenosine, m^2^G = N^2^-Methylguanosine, m^3^C = 3-Methylcytidine).

The 33 analyzed compounds are summarized in Table 2. 23 of the analyzed compounds are resulting from the RNA metabolism, two from the purine metabolism, five from the polyamine/methionine cycle, one from the histidine metabolism and two from the nicotinate and nicotinamide metabolism. Exemplary, the applied MS scan modi are depicted in Electronic Supplementary Material Fig. 2, showing the total ion current (TIC) of the cell culture supernatant of the breast cancer cell line MDA-MB-231 in fullscan with the base peak chromatogram (BPC) of the modified nucleoside N^6^-Threonyl-carbamoyladenosine (t^6^A), the neutral loss scan (−132 Da) with the BPC of t^6^A (m/z 413.2) and product ion scan of t^6^A (m/z 413.2) with the corresponding MS/MS spectrum.

**Figure 3:**
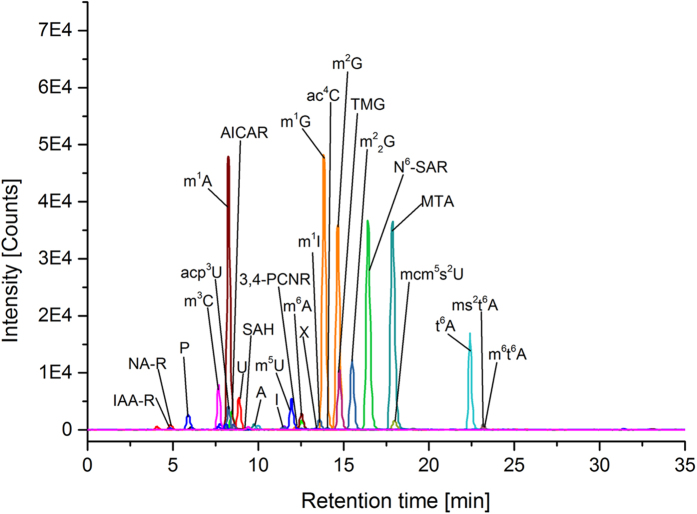
Results of LC-MS-MS analysis of supernatant of breast cancer cell line MDA-MB-453 using MRM operating mode showing MRM transitions of quantifier masses of compounds for semi-quantitative analysis (abbreviations are shown in Table 2).

**Figure 4:**
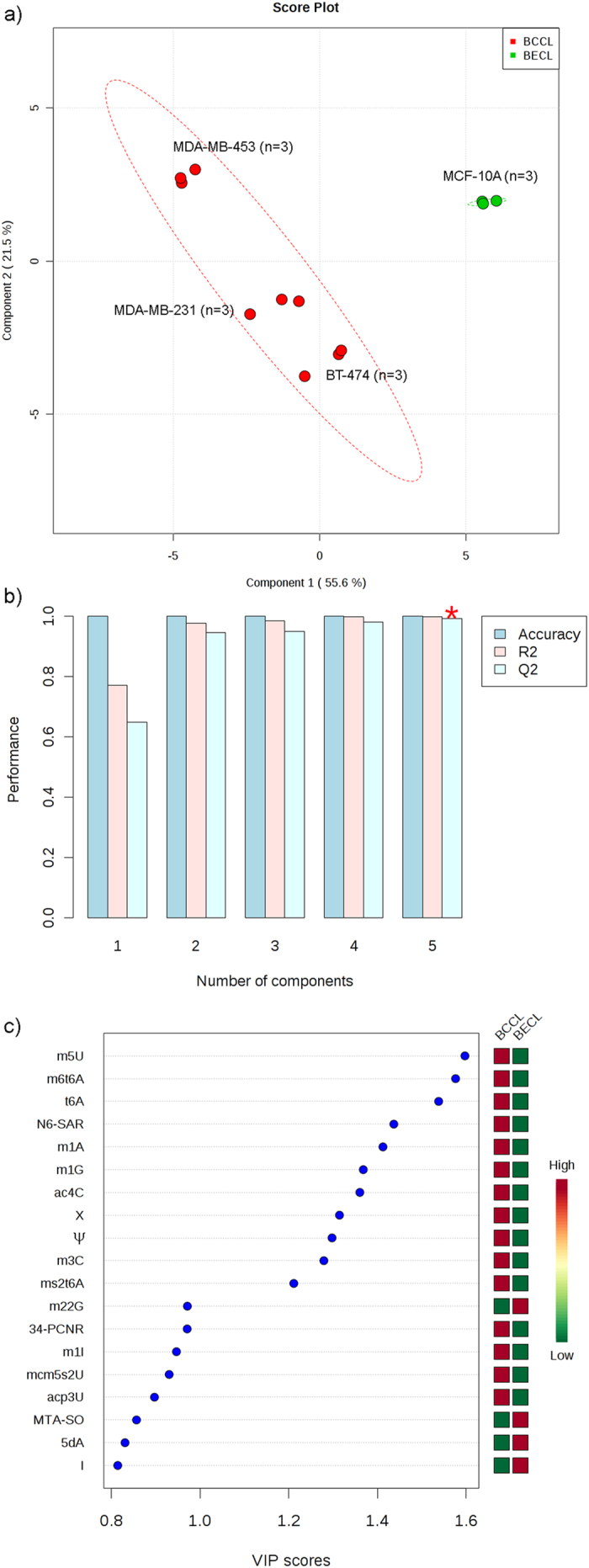
Multivariate analysis of cell lines (**a**) Discrimination of investigated cell lines by PLSDA indicating similarities and differences between samples and groups. Confidence ellipses show 95% confidence regions. Percentage of variance explained by individual component is indicated. (**b**) Performance measurement using different number of components, showing the accuracy, multiple correlation coefficient R^2^ and the explained variance in prediction Q^2^: The red ‘*’ indicates the best value of selected measure (Q^2^). (**c**) Top significant features of metabolites based on VIP score of component 1. Colored boxes indicate the relative concentrations of the corresponding metabolite in each group under study. (BCCL = breast cancer cell line; BECL = breast epithelial cell line).

**Figure 5:**
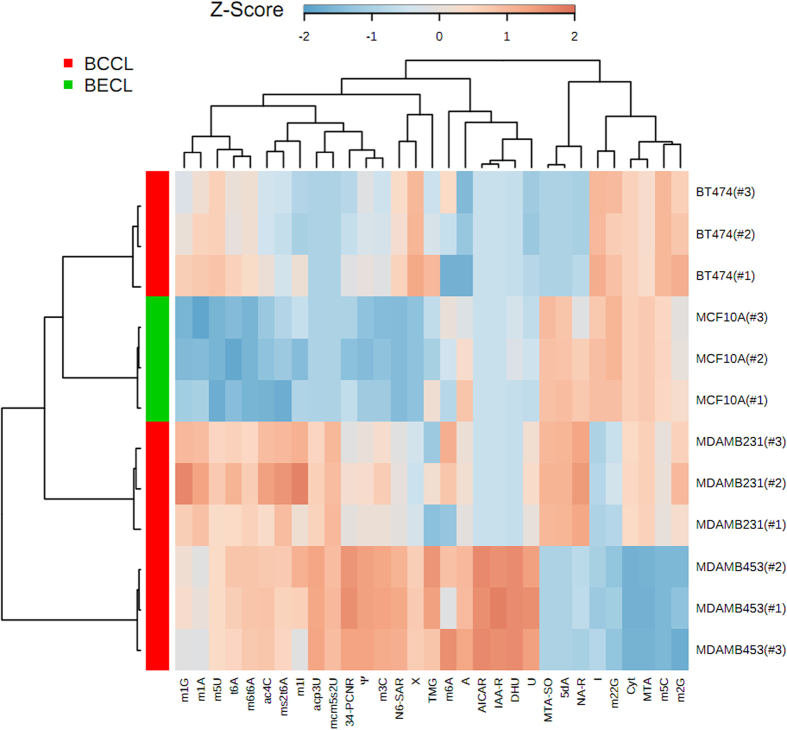
Hierarchical cluster analysis of target compounds detected in breast cancer cell lines (BCCL) MDA-MB-231, -453, BT-474 and breast epithelial cell line (BECL) MCF-10A (n=3). (Rows: samples, columns: target compounds; abbreviations of target compounds are shown in Table 2).

For the comparison of the metabolite excretion of the three breast cancer cell lines with the breast epithelial cell line MCF-10A, we executed a semi-quantitative analysis using MRM operating mode. Therefore we related the peak area of the quantifier mass transitions of each analyte to the peak area of the internal standard isoguanosine. The quantifier MRM transitions of analyzed compounds are shown in Fig. 3. The ascertained values for the compounds in media were subtracted. The resulting values were normalized using the counted number of cells at the moment of supernatant sampling. The cell lines showed significant differences in metabolite excretion (Table 3). Compound levels were generally considered as “elevated” or “decreased”, if the values were a minimum of two standard deviations (2σ-concept) higher or lower than the reference value of MCF-10A. In our semi-quantitative analysis of cell culture supernatants, we observed major differences of metabolite excretion pattern between breast cancer cell lines and MCF-10A, just as well as between the different breast cancer cell lines themselves (Table 3). Breast cancer cell lines could be separated from the reference cell line MCF-10A by PLSDA with component 1 reaching 55.6% variance and component 2 reaching 21.5%, respectively (Fig. 4a). The prediction accuracies have been assessed by cross validation with different numbers of components (Fig. 4b). Q^2^ is a prediction error measure with an optimal value of 1, but Q^2^ can also assume negative values for bad predictive models. Although the best performance could be obtained with five components, a number of two components already delivered an adequate prediction model. The VIP score obtained by PLSDA is an important measure of each independent variable. Higher VIP scores are considered more relevant in classification. The most influential features with a VIP score of 1.2 to 1.6 and moderately influential features with a VIP score of 0.8 to 1.0 are depicted in Fig. 4c. Especially the target compounds m^5^U, N^6^-Methyl-N^6^-threonylcarbamoyladenosine (m^6^t^6^A), t^6^A, N^6^-Succinyloadenosine (N^6^-SAR), m^1^A, m^1^G, ac^4^C, X, Ψ, 3-Methylcytidine (m^3^C) and 2-Methylthio-N^6^-threonylcarbamoyladenosine (ms^2^t^6^A) are elevated, have a high VIP score and should consequently be considered as potential biomarkers for breast cancer. Hierarchical cluster analysis (Fig. 5) of breast cancer cell lines and the breast epithelial cell line MCF-10A revealed that BT-474, followed by MDA-MB-231, is the most similar to MCF-10A based on excretion levels of investigated compounds. The cell line MDA-MB-231 was found to be the most different to the breast epithelial cell line MCF-10A. The differential excretion of metabolites deriving from different biochemical origin will be discussed in the following paragraphs.

### Compounds from RNA metabolism

In contrast to MCF-10A the excretion levels of the modified nucleosides Ψ, m^3^C, m^1^A, m^5^U, m^1^G, X, ac^4^C, t^6^A, ms^2^t^6^A and m^6^t^6^A were elevated in all breast cancer cell lines with Ψ, m^3^C, m^1^A, m^5^U, t^6^A and ms^2^t^6^A showing the most significant differences between breast cancer cell lines and MCF-10A.

The C-glycosidic, modified nucleoside Ψ results from isomerization of specific uridine residues catalized by pseudouridine synthase and has been reported in eukaryotic tRNA, rRNA and snRNA^12^. Urinary levels of Ψ have found to be elevated in breast cancer patients in comparison to healthy subjects^4^,^7^,^35^,^36^,^37^,^38^.

m^3^C, m^1^A, m^1^G, ac^4^C has been reported in eukaryotic tRNA and rRNA^12^. A particular role in directing the cloverleaf folding of tRNA has been attributed to m^1^A, which is methylated post-transcriptionally by methyl-1-adenosine transferase^39^. Elevated amounts of m^1^A have been detected in numerous cancer diseases^40^, as well as in urinary samples of breast cancer patients^35^,^36^,^38^. In comparison to healthy subjects m^1^G has found to be elevated in urinary samples of breast cancer patients^7^,^37^,^38^. It has been reported that m^3^C^41^ and ac^4^C^35^,^37^ are excreted in elevated levels in urinary samples of breast cancer patients, too. m^6^A levels of all breast cancer cell lines showed no significant differences to MCF-10A, because of abnormal standard deviations up to 52%. At this point the transformation of m^1^A into m^6^A under basic conditions, known as the Dimroth rearrangement, has to be taken into concern^42^. In eukarya, m^6^A is the most prevalent methylated nucleoside, accounting more than 80% of all base methylations. The METTL3(methyltransferase like 3)-containing methyltransferase complex catalyzes the methylation of A (Adenosine) to m^6^A immediately after pre-mRNA transcription using SAM as methyl donor. The demethylation of m^6^A is triggered by FTO and ALKBH5 in an iron and α-ketoglutarate-dependant manner. Disbalances in m^6^A have been suggested to be the cause for defects in RNA metabolism. m^6^A in RNA has also been discussed being involved in adipogenesis, spermatogenesis, development, carcinogenesis, stem cell renewal and other unidentified life processes^43^.

The modified nucleosides m^5^U, t^6^A, ms^2^t^6^A have been reported in eukaryotic tRNA^12^. It has been postulated that m^5^U could be involved in the regulation of protein biosynthesis in mammalian liver on the stage of translation^44^. m^6^t^6^A is present in two tRNA species of *Escherichia coli*. The precursor of t^6^A is threonine and the t^6^A synthesis requires ATP and carbonate. The methyl group of m^6^t^6^A originates from methionine. It was found that the methyl group of m^6^t^6^A improves the efficiency of tRNA in *Escherichia coli*^45^. The formation of ms^2^t^6^A occurs via a radical-based post-transcriptional and -translational mechanism at position 37 (A^37^) in tRNAs catalyzed by the enzymes MtaB in bacterial cells and e-MtaB in eukaryotic and archaeal cells, that belong to the methylthiotransferase family of the Radical-SAM enzyme superfamily^46^.

The modified nucleoside D was solely found in MDA-MB-453. D has been reported to occur in tRNA of eukarya^12^, in a total enzymatic digest of nuclear 5S RNA from rat^26^ and in chromosomal RNA of eukarya^47^. Interestingly, it has been reported that the D levels were elevated in urinary samples of breast cancer patients^36^,^38^.

5-Methylcytidine (m^5^C) levels were found to be elevated in BT-474, but decreased in MDA-MB-231 and -453 compared to the reference cell line MCF-10A. m^5^C has been reported to occur in tRNA, rRNA and mRNA of eukarya^12^. m^5^C has been discussed being involved in controlling mechanism for genetic changes in cancer^48^. To the authors’ best knowledge, m^5^C has never been found to be significantly excreted in breast cancer patients.

The levels of the reduced nucleoside 5-Deoxyadenosine (5dA) was elevated in MDA-MB-231, but decreased in MDA-MB-453 and BT-474. In contrast to 5dA, 2’-deoxyadenosine as well as 3’-deoxyadenosine do not contain a cis-diol group and are therefore eliminated during the prepurification by phenylboronic acid affinity chromatography. Consequently they could not be detected by LC-MS measurements. In general, oxidative modification of DNA/RNA is prevalent in cancer, aging and neurodegenerative diseases, leading to the discussion of oxidized nucleosides being a possible biomarker for this phenomenon^49^.

1-Methylinosine (m^1^I) and 5-Methoxy-carbonylmethyl-2-thiouridine (mcm^5^s^2^U) levels were significantly elevated in all breast cancer cell lines, except BT-474. m^1^I and mcm^5^s^2^U occur in tRNA of eukarya^12^. The modified nucleoside m^1^I has been found to be excreted in elevated levels in urinary samples of breast cancer patients^7^,^35^,^37^. Mammalian ALKBH8 (Alkylated DNA repair protein alkB homolog 8), which is involved in mcm^5^s^2^U formation, possesses the tRNA methyltransferase activity that is required for the biogenesis of multiple wobble uridine modifications during translation^50^.

Interestingly, m^2^G levels were elevated in BT-474, but decreased in MDA-MB-453. In MDA-MB-231 m^2^G was slightly, but not significantly elevated. m^2^G occurs in tRNA, rRNA and snRNA of eukarya^12^. In urinary samples of breast cancer patients m^2^G has been reported to be excreted in elevated amounts compared to samples of healthy subjects^7^,^37^.

TMG levels were elevated in MDA-MB-453, in BT-474 and MDA-MB-231 we found no significant differences compared with MCF-10A. TMG has previously been found in mRNA and tRNA of eukarya^12^. To the authors’ best knowledge, TMG has never been reported to be excreted in significantly elevated levels in breast cancer patients.

N^2^-N^2^-Dimethylguanosine (m^2^_2_G) levels were decreased in all breast cancer cell lines, except BT-474. m^2^_2_G occurs in tRNA and rRNA of eukarya^12^. In eukaryotic tRNA m^2^_2_G is present at position 26 in the bend between the D stem and anticodon stem and results from dimethylation catalyzed by human tRNA(m^2^_2_G)dimethyltransferase^51^.

Since Ψ, m^3^C, m^1^A, m^5^U, m^1^G, X, ac^4^C, t^6^A, ms^2^t^6^A and m^6^t^6^A were elevated in all breast cancer cell lines and showed a high VIP score in PLS-DA (Fig. 4), we believe that those compounds could have potential for detection of breast cancer. Subclassification of breast cancer is critical regarding the treatment of individual patients. Differentially excreted nucleosides could be applied for classification of different breast cancer subtypes. In this context, elevated excretion of D and TMG might be characteristic for Her2 overexpression, elevated excretion of m^5^C and m^2^G for Luminal B subtype and elevated excretion of 5dA for Claudin-low subtype, respectively. The origin of RNA seems to play a minor role, because the most striking compounds result from different RNA species. In order to correlate the exometabolom of the investigated cell lines with *in vivo* samples, a variety of human body fluids should be applied for detection of those compounds. As some modified nucleosides could have been correlated with results of *in vivo* samples in the present study, interferences during excretion should be taken into account. Interferences might lead to different nucleoside levels comparing cell lines and *in vivo* samples. Therefore, comparison of cell lines with *in vivo* samples should be regarded with suspicion. We believe that the exometabolom analysis of primary tissue from breast cancer patients and subsequent comparison with cell lines could be promising as a first step.

The unmodified nucleosides C, U and A can be excreted or recycled in the salvage pathway. A can be converted to I catalyzed by adenosine desaminase. The levels of C in RPMI 1640 medium were higher than in supernatants of MDA-MB-453, resulting in negative values. C levels are decreased in all breast cancer cell lines. Accordingly, breast cancer cell lines assimilate C in contrast to MCF-10A. Levels of A were decreased in all breast cancer cell lines, except MDA-MB-453, I levels were decreased in all breast cancer cell lines, except BT-474. Additionally, it has been reported that I occurs in eukaryotic tRNA as a modification of A^52^. U was found to be decreased in BT-474, but elevated in MDA-MB-231 and -453. The nucleoside X was excreted in elevated amounts in all breast cancer cell lines compared with MCF-10A. X has been reported to be increased in urine samples of breast cancer patients, too^26^. The unmodified nucleosides C and U play a central role in the pyrimidine metabolism just as X, I and A in the purine metabolism. Due to the numerous possibilities of metabolization and in due consideration of e.g. the nucleoside salvage, the interpretation of excreted amounts of unmodified nucleosides requires further investigation of the metabolites involved in the purine/pyrimidine metabolism. In consideration of the fact, that different states of cell cycle can be present at the time of sampling and cell counting, the excretion differences of unmodified nucleosides can occur, because these compounds are involved in numerous metabolic pathways and cannot be considered as metabolic end products.

### Compounds from polyamine/methionine cycle

**Figure 6:**
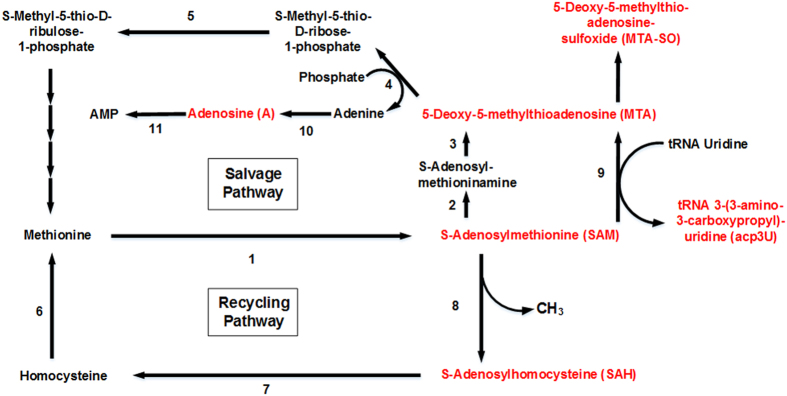
Polyamine/Methionine cycle (red: analyzed compound; involved enzymes: 1) S-adenosylmethionine synthetase [EC:2.5.1.6]; 2) S-adenosylmethionine decarboxylase [EC:4.1.1.50]; 3) spermidine synthase [EC:2.5.1.16]/spermine synthase [2.5.1.22]; 4) 5’-methylthioadenosine phosphorylase [EC:2.4.2.28]; 5) methylthioribose-1-phosphate isomerase [EC:5.3.1.23]; 6) betaine-homocysteine S-methyltransferase [EC:2.1.1.5]/5-methyltetrahydrofolate-homocysteine methyltransferase [2.1.1.13]; 7) adenosylhomocysteinase [EC:3.3.1.1]; 8) DNA (cytosine-5)-methyltransferase 1 [EC:2.1.1.37]; 9) tRNA-uridine aminocarboxy-propyltransferase [EC:2.5.1.25]; 10) adenine phosphoribosyltransferase [EC:2.4.2.7]; 11) adenosine kinase [2.7.1.20]).

In our LC-MS analysis of the different breast cancer cell lines and the breast epithelial cell line MCF-10A we detected the following substances derived from the polyamine/methionine cycle: SAM, SAH, MTA, MTA-SO and 3-(3-Amino-carboxypropyl)-uridine (acp^3^U) (Fig. 6). The elevated methylation of nucleosides in cancer cells may be interrelated with the most popular methyldonor SAM. SAM-synthetase catalyzes the synthesis of SAM from L-Methionine. SAM has found to be elevated in supernatants of MDA-MB-453, in all other cell lines SAM was not detectable. The low excretion patterns of SAM may be explained by the high intracellular utilization of SAM as a methyl donor, e.g. for intracellular nucleoside methylation. In contrast, SAH was elevated in supernatants of all breast cancer cell lines, except BT-474. As SAH is a byproduct of SAM triggered methylation, the elevated excretion might be related to elevated nucleoside methylation in breast cancer cell lines. MTA and MTA-SO excretion levels were decreased in supernatants of all breast cancer cell lines. In the breast cancer cell line MDA-MB-453 the MTA levels of the cell culture medium was higher than the levels of cell culture supernatants. Consequently, MTA was assimilated by the MDA-MB-453 cells. MTA-SO has been found in urine of immunodeficient children and it has been suggested, that MTA-SO results from *in vivo* MTA oxidation by oxides and superoxides or enzymatically by enzymes^53^. The modified nucleoside MTA is part of the methionine metabolism and results from SAM or S-Adenosylmethioninamine. MTA can be converted into S-Methyl-5-thio-D-ribose 1-phosphate catalyzed by 5-Methylthioadenosine phosphorylase (MTAP), thereby Adenine is produced as a byproduct. MTAP is ubiquitously expressed in mammalian tissue and catalyses the first and rate-limiting step of the methionine salvage pathway, resulting in methionine and A. MTAP has been reported to be deficient in breast cancer^54^. Methionine-dependant growth has been discussed in tumor cell lines^55^. Cell lines, that are considered to be methionine-dependant, are unable to grow *in vitro* when methionine is replaced with homocysteine^56^. A relative survival advantage, that would be characteristic for cancer cells, has been suggested for methionine-dependant cell lines^57^. As a result of MTAP deficiency adenine, as well as AMP and A, is solely synthesized in the de novo purine biosynthesis pathway. The decreased excretion of A in BT-474 and MDA-MB-231 might be related with this phenomenon. The depletion of the rate-limiting enzyme MTAP is not responsible for the methionine-dependant growth of human tumor cell lines^56^. It has been observed, that MTA has inhibitory effects on spermidine and spermine synthase and on ornithine decarboxylase. Additionally, MTA influences critical responses of the cell, e.g. regulation of gene expression, proliferation, differentiation and apoptosis^58^. The methyldonor SAM can also be transformed into MTA catalyzed by tRNA-uridine aminocarboxypropyltransferase. Thereby Uridine is transformed into acp^3^U. acp^3^U has been found in phenylalanine tRNA of *Escherichia coli*^59^. As there is no other enzyme reported, we suggest tRNA-uridine aminocarboxypropyltransferase to catalyze acp^3^U synthesis in breast cancer cell lines MDA-MB-231 and -453. Although acp^3^U was slightly elevated in MDA-MB-231 and -453, the MTA levels were decreased in all breast cancer cell lines. Consequently, MTA is further degradaded by MTAP or acp^3^U results from other sources. Another explanation for this phenomenon would be a differential exocytosis between MTA and acp^3^U.

On the basis of MTA and MTA-SO being decreased in supernatants of breast cancer cells, we are assuming that the methionine synthesis is mainly implemented by the recycling pathway. Due to MTA and MTA-SO levels being the highest and SAH levels being the lowest in MDA-MB-231 compared to the other breast cancer cell lines BT-474 and MDA-MB-453, we presume MDA-MB-231 synthesising methionine by the methionine salvage pathway in a higher ratio than the other breast cancer cell lines.

### Compounds from nicotinate/nicotinamide metabolism

1-Ribosyl-imidazolenicotinamide (NA-R) is involved in the nicotinate and nicotinamide metabolism, where it is a product of the reaction of ribose-1-arsenate and niacinamide under elimination of phosphoric acid catalized by the purinenucleoside-phosphorylase [EC: 2.4.2.1] in the cytoplasm. NA-R is a newly discovered precursor of NAD(+) that can be turned into nicotinamide-mononucleotide catalyzed by the nicotinamide ribose kinases Nrk1 and Nrk2 [EC: 2.7.1.22; 2.7.1.173]. Nrk1 is highly specific for the phosphorylation of NA-R as well as the cancer drugs tiazofurin and benzamide ribose. In case of tiazofurin and benzamide ribose the metabolization by Nrk1 results in toxic NAD(+) analogs^60^. Recently it was found that NA-R elevates NAD(+) and increases Sir2 (sirtius deacetylate lysine) functions resulting in improvement of gene silencing, repression of recombination and an extended lifespan without calorie restriction^61^. NA-R was first detected in human urine samples in 1986^62^. As NA-R is elevated in MDA-MB-231 cell culture supernatants and decreased in BT-474 and MDA-MB-453 cell culture supernatants, we suggest that the Nrk1 and Nrk2 activity might be decreased in MDA-MB-231. Due to MDA-MB-231 being a triple-negative, Claudin-low breast cancer cell line, this might indicate a sufficient response to the cancer drugs tiazofurin and benzamide ribose in Claudin-low tumors in contrast to the other investigated subtypes of breast carcinoma. 1-Ribosyl-pyridin-3-one-4-carboxamide (3,4-PCNR) was first isolated and identified in human urine samples in 1979^63^ and is suggested to have his origin in nicotinamidmononucleotide or nicotinamiddinucleotide^64^. 3,4-PCNR has found to be elevated in MDA-MB-231 and -453 cell culture supernatants, but not significantly elevated in BT-474.

### Compounds from histidine/purine metabolism

1-Ribosylimidazole-4-acetic acid (IAA-R) is a metabolic end product of the histidine metabolism with the precursor imidazole-4-acetate, which is the oxidative metabolite of histamine. The biogenic amine histamine results from decarboxylation of the amino acid histidine catalyzed by histidine decarboxylase [EC: 4.1.1.22]. It has been shown, that newly synthesized, nascent histamine is involved in tumor growth of breast cancer patients. The activity of histidine decarboxylase was found to be elevated in breast cancer tissue. Histamine concentrations were significantly decreased in the tumor tissue^65^. IAA-R results from the endogenous ligand imidazole acetic acid ribotide, which is a stimulant of imidazole receptors. Accordingly, IAA-R excretion levels might be correlated with metabolic alterations resulting from cancerous tissue. IAA-R has been solely detected in supernatants of MDA-MB-453. We suggest that excretion levels of IAA-R are not an effect of cancerous alterations of histidine metabolism, but further investigations have to be made to confirm this prediction.

AICAR has been solely detected in supernatants of MDA-MB-453. AICA ribotide is the precursor of AICAR and stimulates the phosphorylation and activation of AMPK resulting in activation of catabolism. Methotrexate and Pemetrexed are drugs, which are used in cancer treatment and directly affect the purine metabolism by inhibiting the IMP formation resulting from AICA ribotide. Liver Kinase B1 (LKB1) activates AMPK and it has been reported that genetic loss in the *LKB1* gene is frequent in cancer. The activated form of AMPK has been discussed as a suppressor of tumorigenesis and inflammation because of its ability to cause cell-cycle arrest and to oppose metabolic alterations occurring in rapidly proliferating cells, like cancerous cells^66^. Supplementation of the cell-permeable AICAR has been reported to increase intracellular levels of AICA ribotide in MDA-MB-231 resulting in impeded lipogenesis, decreased protein translation, blocked DNA synthesis as well as in decreased proliferation, loss of invasive properties and ability of colony formation^67^. El-Masry *et al.* investigated the different effects of AMPK activation by AICA ribotide on different breast cancer cell lines. They found AICA ribotide having anti-proliferative effects on all breast cancer cell lines, but with different sensitivity. In the breast cancer cell line MDA-MB-231 activation of AMPK showed apoptotic effects, but in the breast cancer cell line T47D the same treatment resulted in cell cycle arrest. This led to the presumption that AMPK activation is dependent on the different genetic backgrounds of breast cancer cell lines^68^.

Due to these facts, we presume the elevated excretion of AICAR in MDA-MB-453 to be a result of the breast cancer cell action to avoid the AICA ribotide triggered AMPK activation effects. Therefore, catabolic processes, that are uncharacteristic for tumor cells, are prevented. The correlation between different genetic backgrounds of breast cancer cell lines and the effects of AMPK activation should be further investigated.

AICAR results from AICA ribotide, which is a metabolite from purine biosynthesis and pentose phosphate pathway. Alternatively, AICA ribotide can be further metabolized to inosine monophosphate (IMP) and further on to adenylosuccinate, which is a direct precursor of N^6^SAR. IMP can result from X, G, I and A. The reaction of IMP to adenylosuccinate is catalyzed by adenylosuccinate synthase (EC: 6.3.4.4). Two different isozymes of adenylosuccinate synthase have been separated in rat liver. It has been reported that the acidic isozyme has a lower K_m_ for IMP than the basic isozyme. In rat experimental tissues, human hepatocellular carcinoma and colon adenocarcinoma a isozyme shift to the acidic adenylosuccinate synthase has been observed^69^. With acidic adenylosuccinate synthase having an elevated substrate affinity and being elevated in tumor cells, we suggest the elevated N^6^SAR levels in all breast cancer cell lines in contrast to the breast epithelial cell line MCF-10A being a result of these findings.

## Conclusions

In this study the exometabolom of different cell lines was investigated by LC-MS using a triple quadrupole mass spectrometer. The different subtypes of breast cancer cell lines could be compared to the immortalized breast epithelial cell line MCF-10A under application of a semi-quantitative analysis using MRM. Significant differences in metabolite excretion between MCF-10A and breast cancer cell lines could be observed, but also between the breast cancer cell lines belonging to different breast cancer subtypes. The investigated compounds are all interrelated with biochemical pathways affected by cancerous effects. The gained data shows that the excretion pattern can be utilized as classification model to differentiate between the investigated breast cancer cell lines and the breast epithelial cell line MCF-10A. Especially the metabolic signature consisting of Ψ, m^3^C, m^1^A, m^5^U, m^1^G, X, ac^4^C, t^6^A, ms^2^t^6^A and m^6^t^6^A has great potential for detection of breast cancer, because of the high VIP score in PLS-DA and elevated excretion in all investigated breast cancer cell lines. Correlations between the molecular and genetic profile of different breast cancer subtypes and the excretion pattern should be further investigated, because the metabolic signature of breast cancer cell lines might be a more subtype-specific tool to predict breast cancer, rather than a universal approach. Furthermore, the insights of cell line analysis should be transferred to human body fluids like blood or urine on a following stage, in order to develop a diagnostic system that can be easily applied in clinical praxis. Thereby, a sub classification of breast cancer patients should be taken into account. Due to the complexity of the human metabolism, interferences during the excretion are critical regarding the comparison of *in vivo* samples and *in vitro* model systems, such as cell lines. Consequently, primary tissue should be compared to the insights of the present cell line analysis on a following stage.

## Additional Information

**How to cite this article**: Willmann, L. *et al.* Exometabolom analysis of breast cancer cell lines: Metabolic signature. *Sci. Rep.* **5**, 13374; doi: 10.1038/srep13374 (2015).

## Supplementary Material

Supplementary Information

## Figures and Tables

**Table 1:** Characteristics of investigated breast cancer cell lines.

Cell line	Classification	Immunoprofile	Tissue source	Age (year), race	Histopathological diagnosis	Other Characteristics
MDA-MB-231	Claudin-low	ER^–^, PR^–^, HER2^–^	P	51, C	AC	Ki67, E-cadherin, claudin-3, claudinin-4 and claudinin-7 low. Intermediate response to chemotherapy
MDA-MB-453	HER2	ER^–^, PR^–^, HER2^+^	P	48, C	AC	Ki67 high, chemotherapy responsive
BT-474	Luminal B	ER^+^, PR^+/–^, HER2^+^	O	60, C	IDC	Ki67 high, usually endocrine responsive, variable to chemotherapy.

(P = Pleural effusion, C = Caucasian, AC = Adenocarcinoma, IDC = Infiltrating ductal carcinoma, O = Original tumour, EGFR = Epidermal growth factor receptor, ER = Estrogen receptor, HER2 = Human epidermal growth factor receptor 2, PR = Progesterone receptor)^16^,^70^.

**Table 2:** Compound Setup.

No.	Abbrevation	Compound	[M+H]^+^ (exp.) [m/z]	RT [min]	MRM transitions (Quantifier/Qualifiers) [m/z]	Fragmentor [V]	Collision Potential [V]	Formula	Monoisotopic Mass [Da]	Path- way*
1	SAM	S-Adenosylmethionine	—	3.1	250.4/136.2, 97, 298.1	150	9/25, 25, 9	[C15H23N6O5S]^+^	399.145	P/M
2	IAA-R	1-Ribosylimidazole-4-acetic acid	259.2	4.8	127.1/109.1, 81.1, 54.1	100	10/30, 40, 50	C10H14N2O6	258.085	H
3	NA-R	1-Ribosyl-imidazolenicotinamide	—	4.9	123.1/106.1, 80.1, 78.1	100	10/20, 40, 40	[C11H15N2O5]^+^	255.098	N
4	D	5,6-Dihydrouridine	247.2	5.3	115.2/97.1, 73.2, 55.2	70	5/17, 17, 25	C9H14N2O6	246.085	R
5	Ψ	Pseudouridine	245.2	5.8	209.2/155.2, 125.1, 82.1	100	4/5, 13, 29	C9H12N2O6	244.070	R
6	C	Cytidine	244.2	6	112.2/95.1, 69.2, 67.2	80	5/45, 41, 60	C9H13N3O5	243.086	R
7	m^3^C	3-Methylcytidine	258.2	7.5	126.1/109.1, 95.1, 66.1	100	10/40, 40, 60	C10H15N3O5	257.101	R
8	AICAR	5-Aminoimidazol-4-carboxamid 1-β-D-Ribofuranosid	259.2	7.9	127.2/110.1, 82.2, 55.2	80	5/21, 49, 60	C9H14N4O5	258.096	P
9	m^1^A	1-Methyladenosine	282.2	8	150.2/123.2, 108.2, 94.2	110	13/45, 60, 45	C11H15N5O4	281.112	R
10	acp^3^U	3-(3-Amino-carboxypropyl)-uridine	346.2	8	214.1/197.1, 168.1, 113.1	100	10/20, 20, 40	C13H19N3O8	345.117	P/M
11	m^5^C	5-Methylcytidine	258.2	8.2	126.1/109.1, 108.1, 83.1	100	10/40, 40, 50	C10H15N3O5	257.101	R
12	U	Uridine	245.2	8.7	113.1/96.1, 70.1, 57.1	70	9/33, 33, 25	C9H12N2O6	244.070	R
13	SAH	S-Adenosylhomocysteine	385.2	9.1	135.9/250.1, 133.8, 87.9	130	13/10, 13, 49	C14H20N6O5S	384.122	P/M
14	MTA-SO	5-Deoxy-5-Methylthioadenosine-sulfoxide	314.2	9.3	136.2/119.1, 109.1, 94.1	100	10/20, 30, 40	C11H15N5O4S	313.084	P/M
15	A	Adenosine	268.2	9.5	136.4/119.4, 94.3, 92.3	100	13/49, 49, 60	C10H13N5O4	267.097	R
16	I	Inosine	269.2	11.2	137.3/119.2, 110.2, 55.3	60	4/45, 45, 68	C10H12N4O5	268.081	R
17	m^5^U	5-Methyluridine	259.2	11.7	127.2/110.1, 56.2, 54.2	80	5/33, 45, 52	C10H14N2O6	258.085	R
18	3,4-PCNR	1-Ribosyl-pyridin-3-one-4-carboxamide	271.2	12	139.1/122.1, 94.1	100	10/30, 60	C11H14N2O6	270.085	N
19	5dA	5-Deoxyadenosine	252.2	12.3	136.2/119.1, 92.1, 94.1	100	10/20, 40, 40	C10H13N5O3	251.102	R
20	m^6^A	6-Methyladenosine	282.2	12.3	150.2/123.2, 94.2, 57.2	130	13/49, 49, 45	C11H15N5O4	281.112	R
21	m^1^G	1-Methylguanosine	298.2	12.9	166.2/149.1, 135.1, 110.1	100	9/41, 45, 49	C11H15N5O5	297.107	R
22	X	Xanthosine	285.2	13.2	153.2/136.1, 57.2, 55.2	60	4/33, 25, 25	C10H12N4O6	284.076	R
23	m^1^I	1-Methylinosine	283.2	13.55	151.1/110.1, 94.1, 82.1	80	10/40, 40, 50	C11H14N4O5	282.096	R
24	m^2^G	N^2^-Methylguanosine	298.2	13.65	166.2/149.1, 110.1, 57.2	90	9/37, 45, 60	C11H15N5O5	297.107	R
25	TMG	N^2^,N^2^,7-Trimethylguanosine	326.2	13.65	194.2/149.1, 124.1, 55.2	70	9/45, 45, 68	C13H19N5O5	325.139	R
26	ac^4^C	N^4^-Acetylcytidine	286.2	14.5	154.1/112.1, 95.1, 69.1	80	5/25, 57, 53	C11H15N3O6	285.096	R
27	m^2^_2_G	N^2^-N^2^-Dimethylguanosine	312.2	15.3	180.1/135.1, 110.1, 46.1	100	10/40, 40, 40	C12H17N5O5	311.123	R
28	MTA	5-Deoxy-5-Methylthioadenosine	298.2	16.7	136.2/119.1, 75.2, 61.2	90	13/57, 33, 33	C11H15N5O3S	297.090	P/M
29	N^6^-SAR	N^6^-Succinyloadenosine	384.2	16.9	252.1/162.1, 136.1, 119.1	100	10/40, 30, 60	C14H17N5O8	383.108	P
30	mcm^5^s^2^U	5-Methoxy-carbonylmethyl-2-thiouridine	333.2	17.8	201.1/169.1, 141.1, 82.1	100	10/30, 30, 40	C12H16N2O7S	332.068	R
31	t^6^A	N^6^-Threonyl-carbamoyladenosine	413.2	20.65	281.1/162.1, 136.1, 119.1	100	10/20, 30, 60	C15H20N6O8	412.135	R
32	ms^2^t^6^A	2-Methylthio-N^6^-threonylcarbamoyladenosine	459.2	21.3	327.1/208.1, 182.1, 134.1	100	10/30, 30, 60	C16H22N6O8S	458.122	R
33	m^6^t^6^A	N^6^-Methyl-N^6^-threonylcarbamoyladenosin	427.3	21.35	295.1/150.1, 94.1	100	5/25, 60	C16O8N6H22	426.150	R

(*P/M = Polyamine/methionie cycle; H = Histidine metabolism; R = RNA metabolism; P = Purine metabolism; N = Nicotinate and nicotinamide metabolism).

**Table 3:** Semi-quantitative analysis of cell lines (MDA-MB-231, -453, BT-474) and the breast epithelial cell line MCF-10A.

No.	Compound Abbrev.	MDA-MB-231 (n = 3) AreaQ*1000	±SD	BT-474 (n = 3) AreaQ*1000	±SD	MDA-MB-453 (n = 3) AreaQ*1000	±SD	MCF-10A (n = 3) AreaQ*1000	±SD
1	SAM	n.d.		n.d.		0.28^##^	0.08	n.d.	
2	IAA-R	n.d.		n.d.		4.08^##^	1.30	n.d.	
3	NA-R	97.17^##^	14.92	3.16	0.75	3.83	0.90	27.30	15.08
4	D	n.d.		n.d.		4.89^##^	0.11	n.d.	
5	Ψ	3.05^##^	0.47	2.16^##^	0.34	10.18^##^	0.20	0.65	0.11
6	C	7.23^##^	0.75	19.65^#^	0.88	−7.70^##^	0.54	26.52	3.47
7	m^3^C	26.01^##^	3.87	18.27^##^	1.72	41.36^##^	1.63	10.87	0.89
8	AICAR	n.d.		n.d.		4.51^##^	0.44	n.d.	
9	m^1^A	242.78^##^	28.69	191.26^##^	23.31	146.94^##^	7.55	80.68	15.51
10	acp^3^U	0.25^##^	0.03	n.d.		1.12^##^	0.05	n.d.	
11	m^5^C	1.73^##^	0.18	42.46^##^	1.56	n.d.		7.77	0.26
12	U	8.39^##^	1.49	2.09^#^	0.51	33.23^##^	5.79	3.35	0.42
13	SAH	0.13^##^	0.01	n.d.		0.46^##^	0.01	n.d.	
14	MTA-SO	6.80^#^	0.91	n.d.		n.d.		8.94	4.13
15	A	6.80	0.91	1.53^#^	0.33	17.34^#^	3.10	8.94	4.13
16	I	4.25^##^	0.40	63.38^#^	9.02	3.76^##^	0.86	46.52	5.08
17	m^5^U	29.94^##^	3.56	33.08^##^	3.09	27.33^##^	0.11	6.29	0.35
18	3,4-PCNR	1.51^##^	0.22	1.02	0.24	4.33^##^	0.43	0.72	0.19
19	5dA	52.42^##^	5.13	n.d.		n.d.		22.93	6.06
20	m^6^A	14.06	6.11	9.98	4.14	16.91	5.72	10.28	1.77
21	m^1^G	90.27^##^	12.09	72.01^##^	7.46	71.38^##^	3.77	49.54	3.37
22	X	0.10^##^	0.02	3.59^##^	1.05	1.31^##^	0.09	n.d.	
23	m^1^I	3.36^#^	0.50	2.48	0.30	3.07^#^	0.35	2.30	0.19
24	m^2^G	75.86	9.68	82.36^#^	8.38	34.52^##^	2.34	62.85	4.24
25	TMG	9.70	1.48	11.12	1.53	13.43^#^	0.81	10.15	1.08
26	ac^4^C	7.48^##^	0.92	5.62^##^	0.48	7.28^##^	0.08	4.22	0.23
27	m^2^_2_G	67.93^##^	8.51	154.76	13.11	46.36^##^	3.77	169.73	9.60
28	MTA	23055.89^##^	2263.46	694.37^##^	69.29	−165.91^##^	34.72	98440.31	7508.67
29	N^6^-SAR	0.23^##^	0.03	0.64^##^	0.20	3.20^##^	0.57	n.d.	
30	mcm^5^s^2^U	1.86^##^	0.30	n.d.		1.70^##^	0.13	n.d.	
31	t^6^A	10.14^##^	1.43	8.29^##^	1.13	9.85^##^	0.60	3.93	0.35
32	ms^2^t^6^A	1.21^##^	0.14	0.68^#^	0.03	0.99^##^	0.03	0.53	0.08
33	m^6^t^6^A	0.58^##^	0.08	0.32^##^	0.07	0.84^##^	0.05	n.d.	

Student’s t-test was used to determine the difference of each analyte comparing each breast cancer cell line with the breast epithelial cell line MCF-10A; ^#^:p < 0.05; ^##^:p < 0.01. (AreaQ*1000 = peak area [M+H]^+^ (analyt)/peak area [M + H]^+^ (isoguanosine) *1000 (factor), n.d.: not detectable, SD: standard deviation).
