# Supplementary material for: Exometabolom analysis of breast cancer cell lines: Metabolic signature
Source: Sci Rep. 2015 Aug 21;5:13374. doi: 10.1038/srep13374 (PMC4544000; doi:10.1038/srep13374)

Electronic Supplementary Material

for

Exometabolom analysis of breast cancer cell lines: Metabolic signature

Lucas Willmann[1,4,*]; Thalia Erbes[2,*]; Sebastian Halbach[3, 6, 7]; Tilman Brummer[3]; Markus Jäger[2]; Marc Hirschfeld[8, 9, 10]; Tanja Fehm[5]; Hans Neubauer[5]; Elmar Stickeler[2]; Bernd Kammerer[1,§]

[1] Center for Biological Systems Analysis ZBSA, Albert-Ludwigs-University Freiburg, 79104 Freiburg, Germany
[2] University Medical Center Freiburg, 79106 Freiburg, Germany
[3] Institute of Molecular Medicine and Cell Research, Albert-Ludwigs-University Freiburg, 79104 Freiburg, Germany
[4] Institute of Biology II, Albert-Ludwigs-University Freiburg, 79104 Freiburg, Germany
[5] Universitätsfrauenklinik der Heinrich-Heine-Universität Düsseldorf, Moorenstr. 5, 40225 Düsseldorf, Germany
[6] Institute for Biology III, Faculty of Biology, Albert-Ludwigs-University Freiburg, Germany
[7] Spemann Graduate School of Biology and Medicine, Albert-Ludwigs-University Freiburg, Germany
[8] Department of Obstetrics and Gynecology, University Hospital Freiburg, Freiburg 79106, Germany
[9]German Cancer Consortium (DKTK), Heidelberg, Germany
[10]German Cancer Research Center (DKFZ), Heidelberg, Germany

Contents:

Materials and Methods..1

ESM Figure 1..2

ESM Figure 2..3

Materials and Methods

MRM optimization settings

For purchased standard substances the fragmentor voltage and collision potential for the multiple reaction monitoring (MRM) was determined using the Mass Hunter Optimizer Software (Agilent, Waldbronn, Germany). Thereby the standard substances with a concentration of 0.1 mg/ml were directly injected into the Agilent 6460 triple quadrupol mass spectrometer for 1 minute with a flow rate of 0.5 ml/min using the following solvent composition: 98 % A (water + 0.5 % formic acid) and 2 % B (methanol + 0.5 % formic acid). The injection volume was set to 10 µl, the fragmentor course range was set to 50 - 200 V and the collision energy range was set to 5 – 60 V. The low mass cut-off was set to 50 m/z and the optimization dwell time was 20 ms. Compounds, that were not available as standard substance, were detected in fullscan, neutral loss scan and product ion scan.

Fullscan parameters

For the fullscan the fragmentor voltage was set to 100 V. The mass range was m/z 80-650 with a scan time of 500 ms.

Neutral loss scan parameters

The neutral loss scans were performed at a fragmentor voltage of 100 V and a collision potential of 10 V. The scan time was set to 400 ms. The following masses were used for neutral loss scans: Constant neutral loss (CNL) of 132 amu for unmodified ribose moiety, 162 amu, 178 amu, 249 amu and 263 amu for modified ribose moieties.

Product ion scan parameters

Product ion scans were executed at a fragmentor voltage of 100 V and different collision potentials (5, 10, 20, 30, 40, 50, 60 V). The scan time was set to 300 ms. The mass range was set according to the *m/z* value of the molecular ion and the *m/z* values of the expected fragment ions.

ESM Figure 1: Different Constant Neutral Losses (CNL) indicating decay of alternatively modified ribose moieties

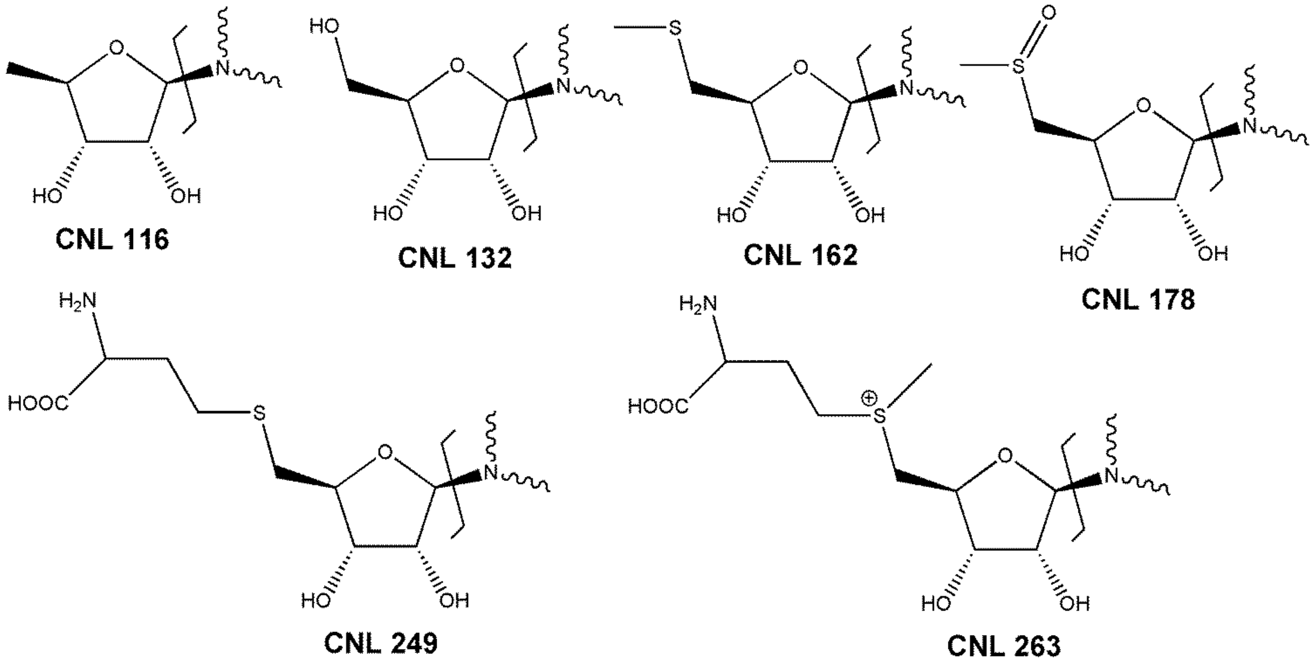

ESM Figure 2: Detection of N⁶-Threonylcarbamoyladenosine (t⁶A) in cell culture supernatants of breast cancer cell line MDA-MB-231 using LC-MS: a) TIC using fullscan; b) BPC of m/z 413.2 using fullscan; c) TIC using neutral loss scan (-132 Da); d) BPC of m/z 413.2 using neutral loss scan (-132 Da); e) BPC of m/z 413.2 using product ion scan (collision potential: 10 V); f) MS/MS spectrum of m/z 413.2

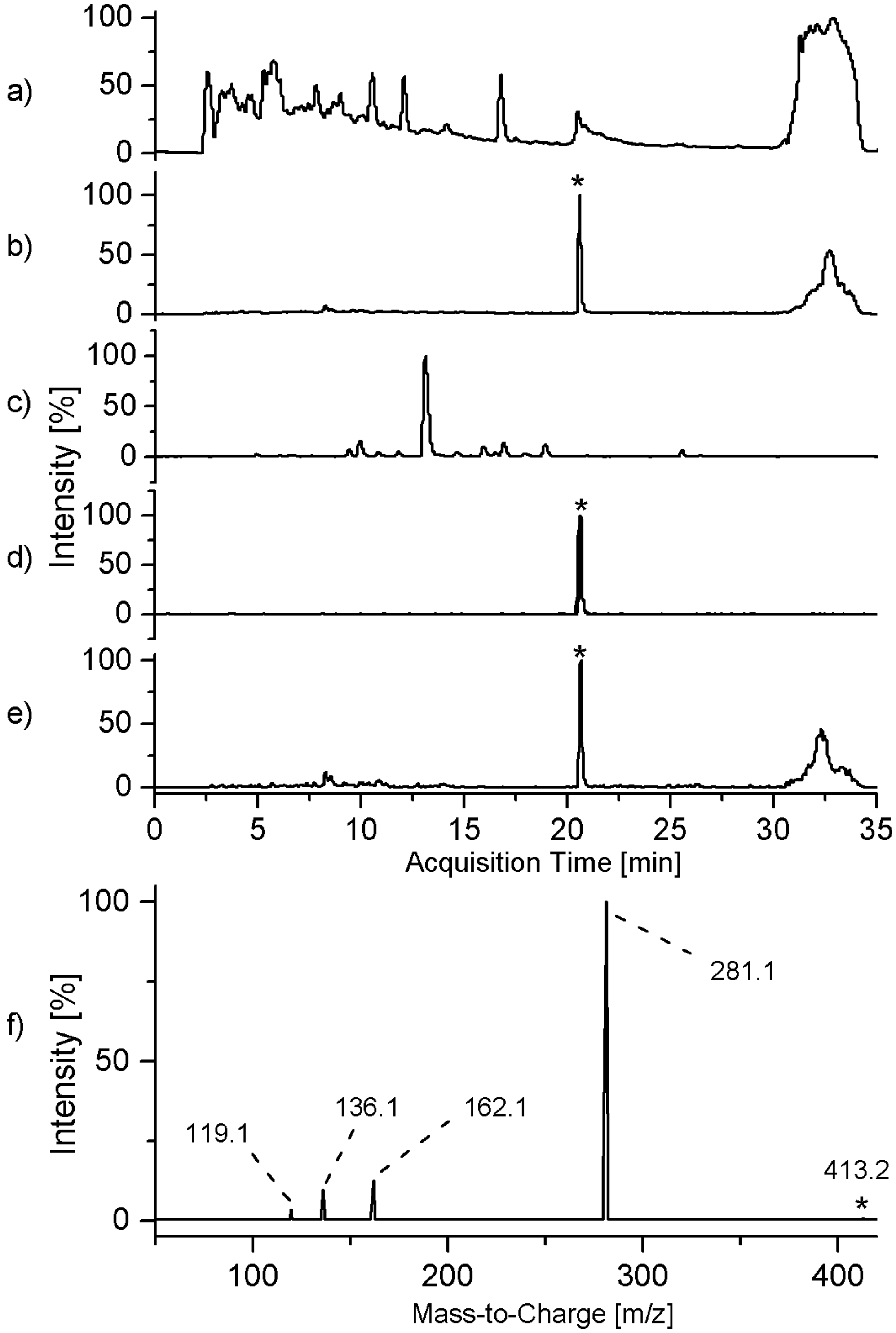

Supplement: Supplementary Information [file srep13374-s1.pdf]
